# A Painless Nodule with Excessive Sweating

**DOI:** 10.4103/0974-2077.53102

**Published:** 2009

**Authors:** BC Sharath Kumar, MG Gopal, AS Nandini, S Praveen Kumar

**Affiliations:** *Department of Dermatology, Venereology and Leprosy, Kempegowda Institute of Medical Sciences, Bangalore, Karnataka, India*

## CASE REPORT

An 18-year-old girl presented with a painless, dusky, raised lesion covered with hairs, over the left shin since two years of her age. The lesion had been slowly growing from the time of appearance and there was history of increased sweating over the lesion. No aggravating or relieving factors for the increased sweating over the lesion could be noted. There was history of frequent trivial trauma to the lesion (due to its location over the anterior shin) associated with serosanguinous discharge and later scab formation.

**Figure 1:**
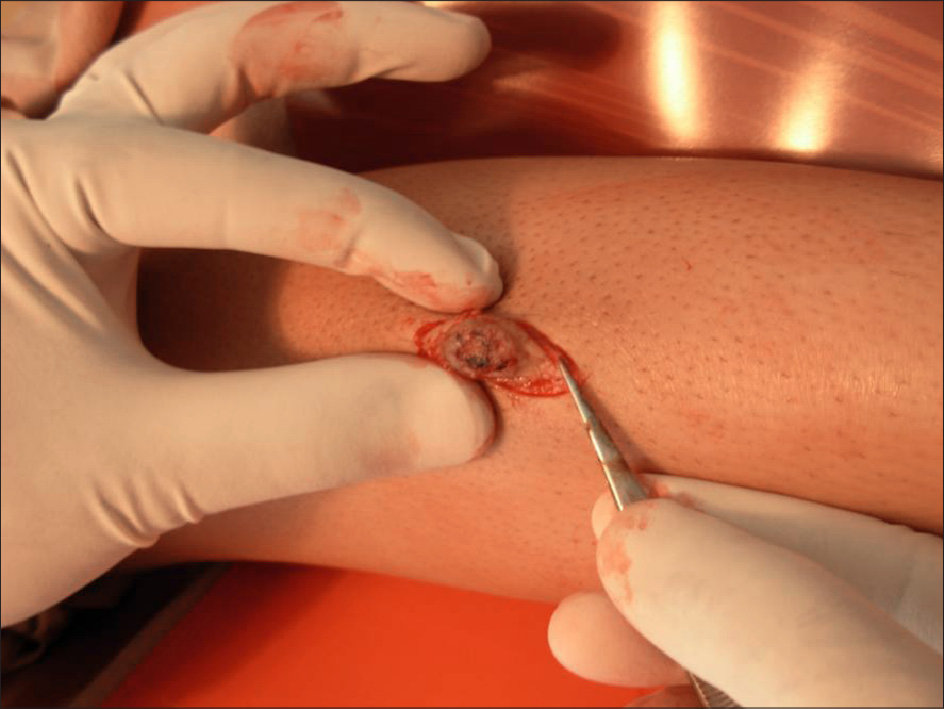
Painless, dusky raised lesion on the shin

**Figure 2:**
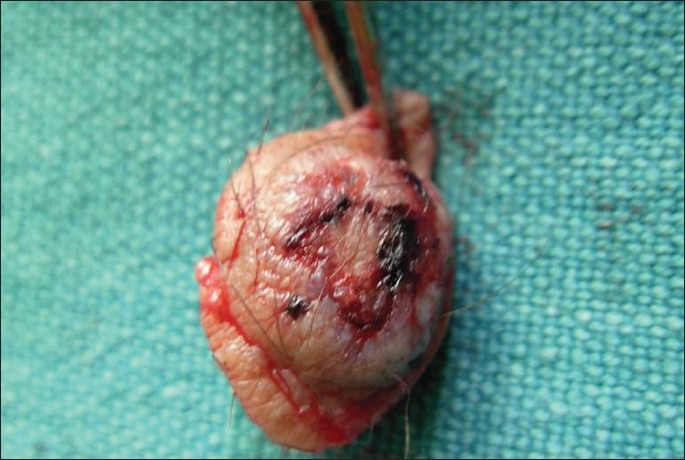
Nodule having terminal hairs and ulcerated surface with scabs

Her general physical and systemic examinations were normal. Cutaneous examination revealed bluish grey, firm, hairy nodule, 2 × 3 cm, in size having an ulcerated surface with scabs at foci over the anterior aspect of the left lower limb [Figures 1 and 2]. Increased sweating and denser, darker coarse terminal hairs were noted over the lesion. Stroking failed to elicit any muscular contraction. No pulsations or bruit could be demonstrated over the lesion.

**Figure 3:**
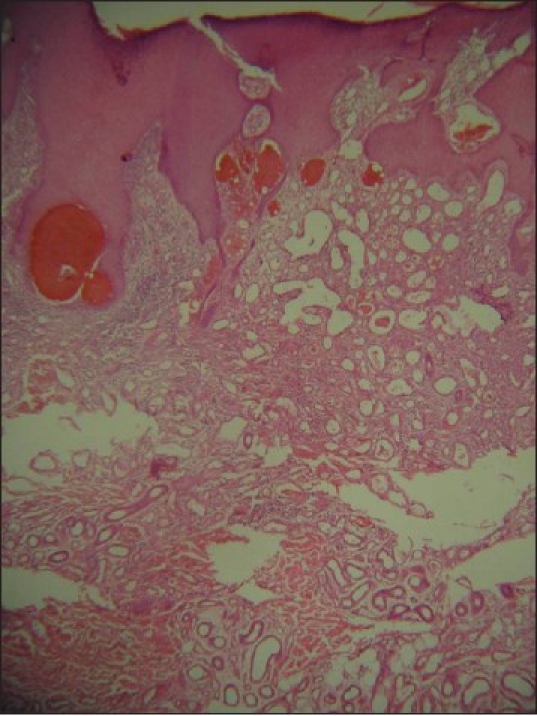
Histopathology reveals increase in blood vessels and eccrine glands in dermis with pseudoepitheliomatous hyperplasia of overlying epidermis (H and E, ×40)

**Figure 4:**
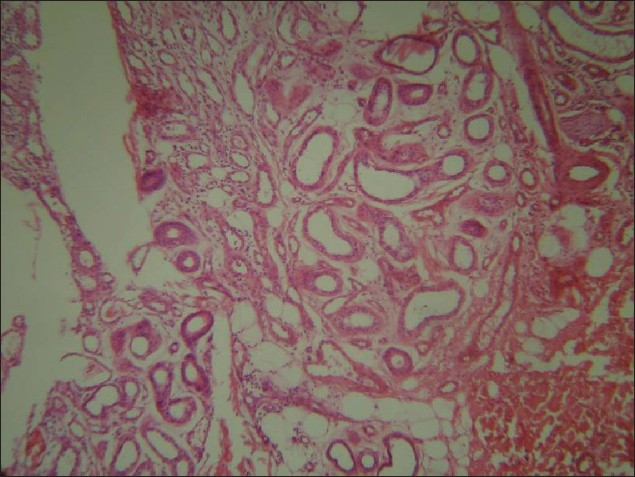
Higher magnification reveals increase in both eccrine glands as well as ducts (H and E, × 100)

Examination of hair, nails and mucous membranes was unremarkable. The lesion was excised and submitted for histopathology [Figures 3 and 4].

## WHAT IS YOUR DIAGNOSIS?

## DIAGNOSIS: ECCRINE ANGIOMATOUS HAMARTOMA

Histopathology showed thickened epidermis and an increased number of small and medium-sized blood vessels [Figure 3] with thin muscular walls of uneven thickness. An increased number of eccrine glands with large coils composed of an increased number of tubules and ducts with a normal proportion of cells were noted in the dermis [Figure 4]. Clinicopathological features supported the diagnosis of eccrine angiomatous hamartoma.

The patient was followed up for two years after excision and there was no recurrence of lesion.

## DISCUSSION

Eccrine angiomatous hamartoma (EAH) is a rare, benign cutaneous tumor usually present at birth or during early infancy and childhood. It has been reported under different names in the literature. Vilanova *et al.,* described an entity called ‘sweating angiomatous hamartoma’ in 1963.[1] It usually presents at birth or during childhood. EAH, though generally asymptomatic, may occasionally present with pain and focal hyperhidrosis (hence the designation sudoriferous angioma).[2–4] The hyperhidrosis is presumably an expression of the presence of the eccrine component.[3,4] Hyman described a similar lesion as EAH.[5] EAH presents as an angiomatous lesion, usually solitary, although cases with multiple lesions have been described.[6]

Histopathologically, EAH is characterized by a dermal proliferation of well-differentiated eccrine secretory and ductal elements closely associated with thin-walled angiomatous channels.[1–4]

On histopathology, EAH is characterized by a dermal proliferation of well-differentiated eccrine secretory and ductal elements closely associated with thin-walled angiomatous channels. Unusual histopathologic variants have been reported and include the infiltration of adipose tissue, increased dermal mucin, and the presence of apocrine glands or pilar structures.[7,8]

In 1971, Zeller and Goldman first described an eccrine pilar angiomatous nevus in a 37-year-old patient[9]. These authors hypothesized that altered chemical interactions between the differentiating epithelium and mesenchyme result in the hamartomatous growth of different elements, creating an abnormal proliferation of vascular and eccrine structures.[9] The lesion in the present case had hairs over it, but did not qualify to be an eccrine pilar angiomatous nevus or hairy EAH as there is no difference in hair density between the lesional and perilesional skin.

The differential diagnoses of EAH include an eccrine nevus, tufted angioma, macular telangiectatic mastocytosis, nevus flammeus, glomus tumor, and smooth muscle hamartoma. These entities can be readily differentiated by histopathology.[4,5] The natural history of EAH is benign and typically slow-growing. Simple excision is usually curative and aggressive treatment is generally unwarranted.
